# Crk and CrkL as Therapeutic Targets for Cancer Treatment

**DOI:** 10.3390/cells10040739

**Published:** 2021-03-27

**Authors:** Taeju Park

**Affiliations:** Children’s Mercy Research Institute, Children’s Mercy Kansas City, Department of Pediatrics, University of Missouri Kansas City School of Medicine, Kansas City, MO 64108, USA; tjpark@cmh.edu

**Keywords:** Crk, CrkL, cancer, cell proliferation, transformation, migration, invasion, epithelial-mesenchymal transition

## Abstract

Crk and CrkL are cellular counterparts of the viral oncoprotein v-Crk. Crk and CrkL are overexpressed in many types of human cancer, correlating with poor prognosis. Furthermore, gene knockdown and knockout of Crk and CrkL in tumor cell lines suppress tumor cell functions, including cell proliferation, transformation, migration, invasion, epithelial-mesenchymal transition, resistance to chemotherapy drugs, and in vivo tumor growth and metastasis. Conversely, overexpression of tumor cells with Crk or CrkL enhances tumor cell functions. Therefore, Crk and CrkL have been proposed as therapeutic targets for cancer treatment. However, it is unclear whether Crk and CrkL make distinct or overlapping contributions to tumor cell functions in various cancer types because Crk or CrkL have been examined independently in most studies. Two recent studies using colorectal cancer and glioblastoma cells clearly demonstrated that Crk and CrkL need to be ablated individually and combined to understand distinct and overlapping roles of the two proteins in cancer. A comprehensive understanding of individual and overlapping roles of Crk and CrkL in tumor cell functions is necessary to develop effective therapeutic strategies. This review systematically discusses crucial functions of Crk and CrkL in tumor cell functions and provides new perspectives on targeting Crk and CrkL in cancer therapy.

## 1. Introduction

Oncogene *v-crk* was identified from chicken tumor virus number 10 (CT10) retrovirus and avian sarcoma virus [1,2]. The cellular counterparts of *v-crk*, CT10 regulator of kinase (*CRK*) and CRK-like (*CRKL*), were discovered in humans on chromosome 17p13.3 and 22q11.21, respectively [3,4,5]. Crk and CrkL are adaptor proteins consisting of SH2 and SH3 domains. CrkII contains a single SH2 domain and two SH3 domains, and its splice variant CrkI lacks the c-terminal SH3 domain [5]. Although CrkI is known to be more effective at inducing cell transformation, it is less clear whether the two Crk splice variants differ in their expression and function. CrkL contains one SH2 and two SH3 domains and is similar to CrkII in structure and function [6]. Human CrkII and CrkL are 57% identical, and mouse CrkII and CrkL are 56% identical in amino acid comparison by the National Center for Biotechnology Information (NCBI) Protein BLAST. CrkII and CrkL are 99% and 97% identical, respectively, between human and mouse, suggesting that both proteins are highly conserved between the two species. Many proteins that bind to the SH2 and the N-terminal SH3 domains of Crk and CrkL have been identified. A few proteins are known to bind to the C-terminal SH3 domain. By mediating protein-protein interactions through their SH2 and SH3 domains, Crk and CrkL play essential roles in signal transduction pathways [7]. Crk and CrkL are expressed ubiquitously, and they have been implicated in diverse biological processes (reviewed by Feller [6] and Birge et al. [8]). Crk and CrkL mediate cytoskeletal changes, cell proliferation, adhesion, migration, differentiation, phagocytosis, and pathogen uptake that are induced by growth factors, tyrosine kinase-coupled receptors, cytokines, integrins, mechanical force, and pathogens. Tyrosine phosphorylation of p130Cas, Cbl, Dab1, FAK, and paxillin play critical roles in these processes.

Due to structural and functional similarities between Crk and CrkL, overlapping roles of both proteins have been studied extensively in many biological processes. Crk and CrkL play essential overlapping roles in neuronal migration, neuromuscular synapse formation, podocyte morphogenesis, T cell migration into inflammation sites, natural killer cell expansion and differentiation during mouse cytomegalovirus infection, lens fiber cell elongation, and postnatal lens capsule development [9,10,11,12,13,14,15]. Furthermore, studies of Crk and CrkL in cultured fibroblasts have revealed that Crk and CrkL play essential overlapping roles in cell structure, motility, and growth [16,17,18]. All these discoveries were made possible by comparing phenotypes resulting from individual and combined knockout of Crk and CrkL in tissues and cells. On the other hand, severe developmental defects of both *CRK*-null and *CRKL*-null mouse embryos indicate that certain developmental processes require Crk or CrkL, or both [19,20]. CrkL has been reported to be essential in Fgf8-induced survival and migration of cells during embryonic development [21]. Moreover, the heterozygous inactive mutation in CrkL in humans has been reported to cause congenital kidney anomalies of DiGeorge syndrome [22]. Recently, Imamoto et al. demonstrated that Crk, like CrkL, genetically interacts with Tbx1 and contributes to the organogenesis affected in DiGeorge syndrome [18]. Interestingly, loss of one allele from both *CRK* and *CRKL* resulted in similar developmental phenotypes to those observed in the loss of two alleles from either *CRK* or *CRKL*, suggesting shared functions by Crk and CrkL. In addition, both protection of *CRK*-null fibroblasts from irremediable endoplasmic reticulum (ER) stress and protection of *CRK*-null or *CRKL*-null kidney cells from toxic insults suggest that both Crk and CrkL mediate stress-induced cell death, with deficiency of one protein making a difference [14,23,24].

Expression of Crk or CrkL is elevated in multiple human cancers, and the overexpression is positively correlated with poor prognosis. Reduced expression of either Crk or CrkL by RNA interference-mediated gene knockdown has been shown to lead to inhibition of in vivo tumor growth of various cancer cell lines. Unlike the systematic and comprehensive approaches made in the field of development, however, many of the early studies of Crk and CrkL in cancer have lacked thorough and quantitative comparisons among phenotypes that resulted from individual and combined gain or loss of Crk and CrkL (Table 1). Consequently, the existing data for Crk and CrkL provide valuable but limited insights into the contribution of Crk and CrkL to cancer cell function. This review systematically analyzes the studies on the role of Crk and CrkL in tumor cell functions to gain perspective on distinct and overlapping functions of Crk and CrkL in cancer cells. For each category of in vitro and in vivo phenotypes, relevant publications have been listed, starting with non-cancer cells and moving to cancer cells. Studies with gene knockdown or knockout are followed by studies with Crk and CrkL overexpression. Furthermore, perspectives on translating scientific knowledge into potential cancer therapy are provided.

## 2. Regulation of the Cytoskeletal Network by Crk and CrkL

Cytoskeletal elements such as microtubules, actin filaments, and intermediate filaments are crucial in determining cell shape and maintaining cell-cell and cell-matrix interactions. Studies of the role of Crk and CrkL in cytoskeletal network rearrangement have provided insights into alterations of the cytoskeleton in cancer cells. Stable overexpression of CrkI in rat 3Y1 fibroblasts resulted in an alteration of cell morphology into refractile and spindle-like shapes [74]. Fibroblasts overexpressing CrkII were slightly spindle-shaped. The degree of the morphological alteration was proportional to the level of CrkI and correlated with colony formation in soft agar and in vivo tumor growth in nude mice. The results indicate that CrkI has a higher transforming capability than CrkII. On the other hand, overexpression of Madin–Darby canine kidney epithelial cells with CrkII or CrkL promoted lamellipodia formation and cell spreading with loss of adherens junctions and reduced β-catenin staining at cell-cell junctions [75]. Overexpression of T47D breast cancer epithelial cells with CrkII also caused dispersal of colonies [75]. CrkII overexpression in NIH3T3 cells made cells larger with enhanced lamellipodium formation [76]. In addition, overexpression of non-small cell lung cancer cells with CrkI or CrkII decreased expression of p120-catenin, a key member of adherens junction [77]. These studies demonstrate that overexpression of Crk family proteins results in reorganization of the actin cytoskeleton, weakened cell-cell junctions, and enhanced lamellipodia or spindle formation, thus leading to enhanced dispersal and spreading of cells.

On the other hand, gene knockdown studies have provided different insights into the functions of Crk and CrkL on the cell cytoskeleton. Crk knockdown in NIH3T3 cells resulted in a slower rate of cell spreading, more filopodia formation, and reduced focal adhesion formation in the early stages of cell spreading onto fibronectin-coated surfaces [76]. Furthermore, Crk/CrkL double knockdown, but not Crk or CrkL single knockdown, in NIH3T3 cells led to nearly complete inhibition of focal adhesion formation in the absence of PDGF [78]. The Crk/CrkL double knockdown also inhibited platelet-derived growth factor (PDGF)-induced circular dorsal membrane ruffle formation as well as focal adhesion disassembly. CrkII and CrkL preferentially activated the small GTPase Rac1, whereas CrkI preferentially activated Rap1 [78]. In addition, ablation of both Crk and CrkL from mouse embryonic fibroblasts resulted in a decrease in focal adhesion sites, reduced actin stress fibers, and a collapse of microtubule structures [16]. Fibroblasts lacking Crk or CrkL alone displayed a much more modest phenotype. In addition, breakdown of adherens junctions between neighboring fibroblasts was inhibited in immortalized mouse embryonic fibroblasts lacking both Crk and CrkL, leading to failure of cell dispersion and tight cell cluster formation [17]. These results suggest that Crk and CrkL have essential overlapping functions in maintaining the cytoskeletal network integrity and controlling cytoskeletal network rearrangement in fibroblasts.

The effect of Crk and CrkL on the cell cytoskeleton has also been demonstrated in cancer cell lines. Crk knockdown resulted in decreased spreading of a breast cancer cell line onto a fibronectin substrate with decreases in focal adhesions and actin stress fibers [25]. In addition, knockdown of both Crk and CrkL in the breast cancer cell line resulted in defective lamellipodia formation and delayed cell spreading on fibronectin [26]. In a glioblastoma cell line, both CrkL knockdown and Crk/CrkL double knockdown caused cells to shrink and become rounded. The morphological alterations, quantified by the cytoplasmic area and the roundness, were more significant with Crk/CrkL double knockdown than with CrkL knockdown [27]. Taken together, these studies demonstrate that Crk and CrkL play critical overlapping roles in the reorganization of the cytoskeletal network and are required for the spreading of fibroblasts and cancer cells.

## 3. Regulation of In Vitro Cell Proliferation by Crk and CrkL

Uncontrolled cell proliferation is a crucial feature of many tumor cell lines. Crk and CrkL have been studied for their roles in normal and cancer cell proliferation. A close comparison of the Crk- and CrkL-mediated effects between normal and cancer cells may provide valuable insights into therapeutics. Ablation of endogenous Crk and CrkL from immortalized fibroblasts using Cre-loxP recombination-mediated gene knockout caused a blockage of cell proliferation and arrest of the cell cycle at the G1-S transition [17]. While loss of either Crk or CrkL alone conferred a much more modest reduction in cell proliferation, reintroduction of CrkI, CrkII, or CrkL individually rescued cell proliferation in the absence of the endogenous Crk and CrkL, suggesting overlapping functions of Crk and CrkL in fibroblast proliferation.

While there are few reports about the effects of Crk and CrkL on cell proliferation in non-transformed cells, Crk and CrkL have been studied extensively for their effects in a number of cancer cell lines. In those studies, Crk and CrkL were ablated by gene knockdown or overexpressed to investigate their cellular functions. Reduction in CrkII and CrkI protein levels by Crk knockdown led to inhibition of cell proliferation in MCAS and SKOV3 ovarian cancer [28,29] and synovial sarcoma cell lines [30] (Table 1). CrkL knockdown also inhibited cell proliferation in rhabdomyosarcoma [33], MDA-MB-453 breast cancer [34], MKN-45 gastric cancer [35], hepatocellular carcinoma [39], and HeLa cell lines [36]. Loss of both Crk and CrkL by CRISPR/Cas9 caused inhibition of proliferation of colon cancer cells [40]. In addition, individual and combined knockdown of Crk and CrkL in U-118MG glioblastoma cells demonstrated that CrkL knockdown and Crk/CrkL double knockdown, but not Crk knockdown, inhibited cell proliferation [27].

On the other hand, CrkII overexpression promoted proliferation of Hca-P hepatocarcinoma cells [31]. CrkL overexpression also promoted cell proliferation in non-small cell lung cancer (NSCLC) [32], cervical carcinoma [37], and Ishikawa endometrial carcinoma [38] cell lines. Crk knockdown and CrkL knockdown induced arrest of the cell cycle at G1 in synovial sarcoma [30] and gastric cancer cells [35], respectively. In contrast, cell cycle progression was observed with CrkL overexpression in NSCLC [32] and Ishikawa endometrial carcinoma [38] cell lines.

Taken together, these studies demonstrate essential functions of Crk or CrkL in cell cycle progression and cell proliferation in a variety of cancer cell lines. Since many of these studies addressed either Crk or CrkL, but not both, it is less clear whether the effects on cell proliferation are unique to one protein or shared by both proteins in a given cancer type. Therefore, it is premature to conclude why some cancer cells depend on Crk and others depend on CrkL to proliferate. Systematic comparison among individual and combined knockdown in various cancer cell lines would provide in-depth insights into this question.

## 4. Regulation of Cell Transformation by Crk and CrkL

v-Crk was discovered as an oncogenic fusion protein that lacks catalytic activity but contains the viral gag sequence followed by an SH2 and an SH3 domain [1]. v-Crk was later found to be similar to CrkI [3]. Overexpression of v-Crk induced increases of tyrosine-phosphorylated proteins and its association with phosphotyrosine-containing proteins, leading to transformation of chicken embryonic fibroblasts [1,79,80]. Both SH2 and SH3 domains were required for the v-Crk-induced transformation [81].

Matsuda et al. [74] cloned human *CRKI* and *CRKII* and demonstrated that CrkI overexpression in rat 3Y1 cells induced transforming activities such as spindle-like cell morphology, anchorage-independent cell growth, and in vivo tumor growth in nude mice. However, cells with CrkII expression did not exhibit significant transforming activities except for slight morphological changes. On the other hand, CrkII overexpression induced transformation in different cell types, including Rat-1 [82] and NIH3T3 [83] fibroblasts. CrkL overexpression also induced cell transformation in Rat-1 fibroblasts in a RAS-dependent manner [84] and immortalized human airway epithelial cells [85]. Zheng et al. [86] demonstrated that C3G and SOS1 are required for anchorage-independent growth of NIH3T3 cells upon CrkI overexpression. In addition, Dok1 and RasGAP are reported to mediate Abl inhibition-mediated enhancement of transformation of NIH3T3 cells upon CrkI overexpression [87]. Furthermore, oncogenic transformation induced by *v-fos* or *v-ras* infection was inhibited in mouse fibroblast cell lines lacking endogenous Crk or CrkL [88].

Contribution of Crk and CrkL to anchorage-independent growth was demonstrated in several cancer cell lines (Table 1). Crk knockdown in human ovarian cancer cells and glioblastoma cells inhibited colony formation on soft agar [28,41]. CrkL knockdown also inhibited colony formation of glioma and cervical cancer cell lines on soft agar [42,43]. Ablation of CrkII, CrkI, and CrkL altogether suppressed anchorage-independent growth of MDA-231 breast cancer cells [26]. On the other hand, CrkII overexpression increased colony formation on soft agar in murine hepatocarcinoma cells [31]. These studies together suggest that overexpression of Crk or CrkL induces transformation of fibroblasts and that both Crk and CrkL are required for transformation of tumor cells. Since the transforming activities of CrkII, CrkI, and CrkL were not compared in each cell type, it is less clear whether different cell types require different Crk proteins for transformation.

## 5. Regulation of In Vivo Tumor Growth and Metastasis by Crk and CrkL

In line with their contribution to in vitro cell proliferation and anchorage-independent cell growth, effects of Crk and CrkL on in vivo tumor growth have been studied using the gene knockdown or knockout approach (Table 1). Crk knockdown in ovarian cancer [28], glioblastoma [41], breast cancer [26,44], and bladder cancer [45] cells resulted in decreased tumor growth in nude mice. CrkL knockdown also inhibited in vivo tumor growth in head and neck squamous cell carcinoma [46], rhabdomyosarcoma [33], hepatocellular carcinoma [39], and colorectal cancer [47] cells.

In addition to suppressing tumor growth at the primary injection sites in nude mice, Crk or CrkL knockdown reduced metastatic tumor burden. Bone metastasis in vivo following intra-cardiac injection of basal breast cancer cells was reduced by Crk knockdown [26]. Crk knockdown reduced metastatic tumor burden in blood, liver, and lung together with reductions in tumor volume at the primary site and the number of circulating tumor cells following orthotopic injection of bladder cancer cells under the bladder muscle layer [45]. In addition, Franke et al. [47] reported that SASH1 deficiency in colon cancer cells increased in vivo tumor formation at the primary site and the number of metastatic lesions, the latter being blocked by CRISPR/Cas9-based CrkL deficiency. These results demonstrate that in vivo tumor growth, likewise in vitro tumor cell proliferation, depends on Crk and CrkL.

## 6. Regulation of Cell Migration and Invasion by Crk and CrkL

Park and Curran reported that neuronal progenitor cell-specific knockout of *CRK* and *CRKL* was induced in mice to demonstrate that Crk and CrkL play essential overlapping roles in Reelin-dependent neuronal migration in the developing brain [9]. In this study, mice lacking Crk or CrkL alone in neurons did not exhibit the failure in neuronal migration. This was the first in vivo study to demonstrate the essential overlapping roles of Crk and CrkL in cell migration. Functions of Crk and CrkL in cell motility have been further studied at the cellular level. Antoku and Mayer [78] reported that PDGF-induced motility of NIH3T3 cells was decreased with Crk knockdown or CrkL knockdown and completely blocked with Crk/CrkL double knockdown. Ablation of both Crk and CrkL in mouse embryonic fibroblasts resulted in a decrease in cell motility [16,17]. Mouse fibroblasts lacking CrkL exhibited a decrease in cell migration toward fibronectin but not toward high serum [89]). In addition, T cells lacking both Crk and CrkL showed defects in in vitro cell migration to CCL21 and CXCL10, trans-endothelial migration, and in vivo cell migration to inflamed tissues [11]. In contrast, Crk knockdown enhanced endothelial cell migration under intermittent hypoxia [90]. Overexpression of CrkII promoted cell migration and invasion in COS-7 cells [91,92] and Crk-null fibroblasts [56]. Expression of CrkII with a mutated SH2 domain inhibited insulin-induced migration and invasion of COS-7 cells [91,92]. CrkL overexpression promoted spontaneous migration of Ba/F3 hematopoietic cells [93].

Effects on cancer cell migration and invasion are the most extensively studied function of Crk and CrkL (Table 1). Rodrigues et al. [25] reported that Crk knockdown led to inhibition of cell migration and invasion in breast cancer, cervical carcinoma, and non-small cell lung carcinoma. Furthermore, Crk knockdown inhibited hepatocyte growth factor (HGF)-induced migration of breast cancer cells. Since then, Crk knockdown has been demonstrated to result in decreases in cancer cell migration and invasion in ovarian cancer [28,29], synovial cell carcinoma [52], glioblastoma [41], oral squamous cell carcinoma [53], prostate cancer [54,55], breast cancer [26], gastric cancer [59], bladder cancer [45], and pancreatic ductal adenocarcinoma [60] cells. CrkL knockdown also inhibited cell motility in head and neck squamous cell carcinoma [46] and migration and invasion of cervical cancer cells [43]. Furthermore, CrkL knockdown led to inhibition of TGF-β1-induced cell motility in glioma [42] and ovarian cancer [61] cells. CrkL knockdown also resulted in a decrease in CCL20-induced migration and invasion of gastric cancer cells [63]. However, it was not clear whether the other protein between Crk and CrkL play similar roles because all the studies investigated effects by either Crk knockdown or CrkL knockdown in the given cancer cell type.

Recently, Franke et al. [40] demonstrated that loss of Crk or CrkL alone using CRISPR/Cas9 substantially inhibited migration and invasion of colorectal cancer cells, while loss of both Crk and CrkL completely blocked cell migration and invasion. Crk/CrkL double knockout also inhibited pancreatic cancer cell migration and invasion. To investigate distinct and overlapping functions of Crk and CrkL, individual and combined knockdown of Crk and CrkL were induced in U-118MG glioblastoma cells, and cell migration and invasion were analyzed in real-time. While CrkL knockdown reduced glioblastoma cell migration, Crk knockdown delayed the cell migration [27,94]. When knockdown of both Crk and CrkL was induced, cell migration was completely blocked, suggesting the unique and overlapping functions of Crk and CrkL in glioblastoma cells. On the other hand, CrkL knockdown, but not Crk knockdown, inhibited glioblastoma cell invasion, and Crk/CrkL double knockdown completely blocked cell invasion. These findings indicate that individual and combined ablation of Crk and CrkL are required to address their unique and overlapping functions in tumor cells. In addition, real-time monitoring of cell migration and invasion might be needed to fully understand the contributions of Crk and CrkL to cell migration and invasion [94].

The effect of Crk and CrkL on tumor cell migration and invasion has also been studied using overexpression of Crk or CrkL in tumor cells. Transfection of U87MG glioblastoma cells with CrkI, but not CrkII, increased cell migration and invasion [49]. CrkII overexpression led to increased migration of rat bladder carcinoma cells [48], increased motility of fibrosarcoma [51], increased migration and invasion of Hs683 glioblastoma [50], increased migration of breast adenocarcinoma [56], and increased migration and invasion of murine hepatocarcinoma [31] cells. In contrast, expression of the N-terminal SH3 domain of Crk reduced motility and invasion of NSCLC cells [57]. Whereas CrkL overexpression inhibited migration and invasion of murine hepatocarcinoma cells [62], CrkL overexpression promoted invasion of cervical carcinoma cells [37].

The roles of tyrosine and serine phosphorylation of CrkII in binding to its target proteins in relation to cell motility have also been studied. Noren et al. [95] reported that treatment of breast cancer cells with Ephrin-B2 Fc increased CrkII phosphorylation on Tyr221 in an Abl-dependent manner, leading to inhibition of cell motility and invasion. Both CrkII-Y221F expression and Crk knockdown blocked the Ephrin-B2 Fc-induced inhibition of cell migration. Stimulation of HeLa cells with HGF also induced phosphorylation of CrkII on Tyr221 in an Abl-dependent manner, which led to inhibition of HGF-induced cell migration and served as a negative feedback loop [96]. Abl-induced phosphorylation of CrkII on Tyr221 and subsequent intramolecular binding of the SH2 domain to the phosphorylated Tyr221 are known to disrupt the association of CrkII to its SH2 target proteins [97,98]. In addition, Kobashigawa et al. [99] demonstrated that the inter-SH3 linker binds to SH3 domains and regulates the association of the N-terminal SH3 domain to its targets. Therefore, these two kinds of intramolecular binding within CrkII reduce its transforming ability compared to CrkI.

Whereas overexpression with CrkII-Y239F, like wild-type CrkII, increased migration of Crk-null fibroblasts and Hs683 glioblastoma cells, CrkII-Y239F, unlike wild-type CrkII, failed to increase migration of 4T1 breast cancer cells [56]. Phosphorylation of CrkII on Tyr251 that is located on the C-terminal SH3 domain promoted motility of Crk-null mouse fibroblasts towards epidermal growth factor (EGF) [100]. On the other hand, CrkII-Ser41Gly expression decreased motility of NSCLC cells while wild-type CrkII expression increased the cell motility, suggesting that phosphorylation of CrkII on serine 41 by PAK1 promotes cell motility and invasion [58]. Therefore, depending on cancer cell types, phosphorylation of CrkII on different residues appears to be induced in response to external stimuli to modulate cell motility.

**Figure 1:**
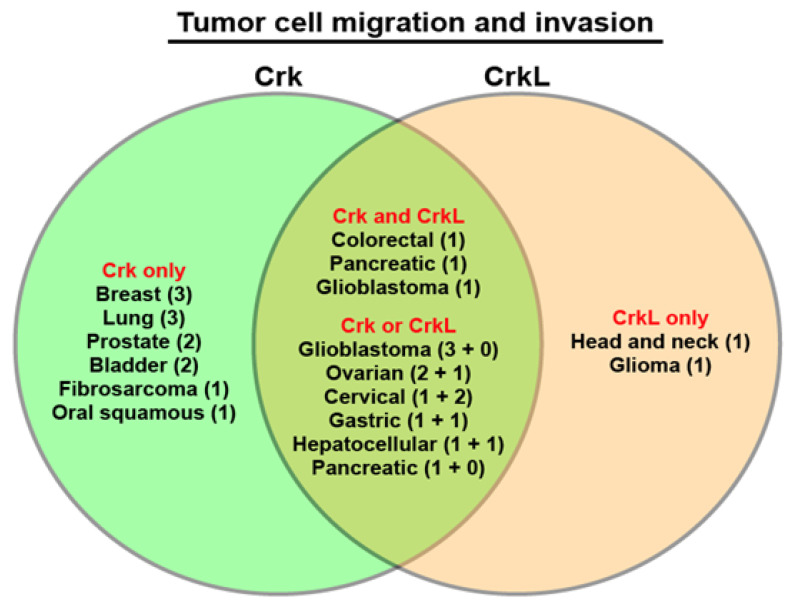
Diagram of cancers that are dependent on Crk and CrkL for tumor cell migration and invasion. The studies of Crk and CrkL in tumor cell migration and invasion were sorted by distinguishing whether Crk or CrkL or both were investigated in a given study. Then the studies were grouped into cancer types. The numbers inside the parentheses indicate the number of publications. The group “Crk and CrkL” includes the studies in which individual and combined alterations of Crk and CrkL expression were conducted at the same time, and the results were compared. The group “Crk or CrkL” includes the studies in which an individual alteration of Crk or CrkL expression was conducted in multiple publications. The two numbers for the group “Crk or CrkL” indicate the number of publications for Crk plus the number of publications for CrkL. For example, glioblastoma has 3 publications for Crk and 1 publication for both Crk and CrkL.

All these results suggest that Crk and CrkL play critical roles in tumor cell migration and invasion in a variety of cancers. In most of these studies, loss of either Crk or CrkL resulted in decreases in tumor cell migration and invasion, suggesting that tumor cell migration and invasion are highly demanding cellular processes that require both Crk and CrkL to reorganize the cellular cytoskeletal network. In particular, both Crk and CrkL have been demonstrated to be important for tumor cell migration and invasion in colorectal cancer, glioblastoma, ovarian cancer, cervical cancer, gastric cancer, and hepatocellular carcinoma (Figure 1). Furthermore, recent studies indicate that cell migration and invasion depend on Crk and CrkL in a dose-dependent manner since the loss of both Crk and CrkL led to more severe defects than the loss of one protein. In addition, the complete blockage of cell migration and invasion in the absence of Crk and CrkL suggests that cancer cells depend entirely on Crk and CrkL for their motility. Taken together, these results demonstrate that cell motility is a genuine cellular function of Crk and CrkL.

## 7. Regulation of EMT and Chemoresistance by Crk and CrkL

The epithelial-mesenchymal transition (EMT) is a process by which epithelial cells lose their cell polarity and cell-cell adhesion, and gain mesenchymal cell properties such as spindle shape, increased survival, and migratory and invasive properties. At the molecular level, EMT is characterized by loss of epithelial markers, including E-cadherin, integrins, and cytokeratins, and gain of mesenchymal markers, including N-cadherin, vimentin, and fibronectin. The EMT switch is mediated through transcriptional reprogramming of crucial transcription factors such as Snail, Slug, TWIST1, ZEB1, and ZEB2 [101]. Knockdown of CrkI and CrkII significantly reduced the EMT in human bladder cancer cells by decreasing expression of N-cadherin, ZEB-1, vimentin, and fibronectin and increasing E-cadherin expression. This effect was accompanied by a reduction in HGF expression, c-Met activity, and Gab1 phosphorylation [45] (Table 1). Crk knockout using CRISPR/Cas9 decreased N-cadherin expression and increased expression of β-catenin and E-cadherin in murine breast adenocarcinoma cells, leading to loss of mesenchymal-like spindle-shaped cell morphology and increased cell-to-cell adhesion [44].

CrkL knockdown also suppressed CCL19/CCR7-induced EMT marker expression and extracellular signal-regulated kinase (ERK) phosphorylation in epithelial ovarian carcinoma cells by decreasing expression of N-cadherin, Snail, and MMP9 and increasing E-cadherin expression [65]. CrkL knockdown inhibited CCL20/CCR6-induced EMT marker expression and ERK phosphorylation in gastric cancer cells by decreasing expression of N-cadherin, vimentin, and MMP2 [63]. While CrkL knockout blocked SASH1 deficiency-induced EMT in HCT116 colon cancer cells [47], Crk/CrkL double knockout inhibited Src activation-induced EMT [40]. Increased E-cadherin expression and decreased ZEB1 expression were observed by knockout of Crk, CrkL, or both in colon cancer cells and by Crk/CrkL double in pancreatic cancer cells [40]. On the other hand, overexpression of A549 human lung carcinoma cells with CrkI or CrkII stimulated the EMT by increasing expression of Snail, Slug, N-cadherin, Fibronectin, and MMP2 and decreasing E-cadherin expression [64]. The EMT induction by CrkI or CrkII overexpression was accompanied by increased TGF-b1 expression and mediated by Rac1 and RhoA. These results suggest that cells require Crk and CrkL for EMT.

Two in vivo studies have suggested a link between EMT and chemoresistance. Zheng et al. [102] demonstrated that suppression of EMT in pancreatic ductal adenocarcinoma mouse models by genetic ablation of EMT-inducing transcription factors such as Snail or Twist enhanced expression of nucleoside transporters in tumors, leading to enhanced sensitivity to gemcitabine treatment and increased overall survival of mice. In addition, Fischer et al. [103] demonstrated that breast cancer cells with EMT contribute to recurrent lung metastasis after chemotherapy, and that inhibition of EMT by overexpressing miR-200 abrogated the chemoresistance to cyclophosphamide treatment, leading to reduced lung metastasis. Interestingly, CrkL overexpression led to increased chemoresistance to cisplatin treatment in cervical carcinoma [37] and endometrial carcinoma [38] cells. Recently, Franke et al. [40] demonstrated decreased chemoresistance and EMT inhibition by loss of both Crk and CrkL in colon cancer cells. These results suggest that Crk and CrkL play important roles in EMT and chemoresistance and may provide a therapeutic target to improve the efficacy of chemotherapy drugs.

## 8. Overexpression of Crk and CrkL in Human Cancers and Lower Survival

**Figure 2:**
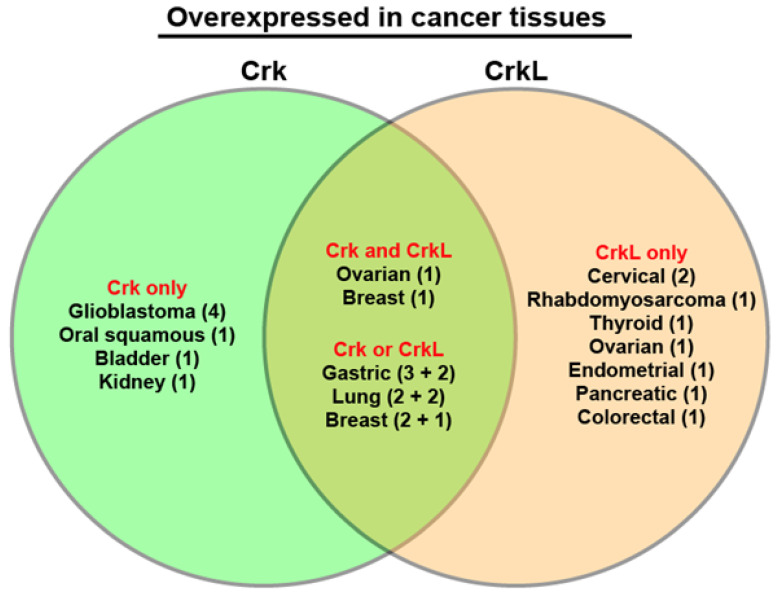
Diagram of cancers in which Crk and CrkL are overexpressed. The studies of Crk and CrkL protein expression in cancer tissues were sorted by distinguishing whether Crk or CrkL or both were investigated in a given study. Then the studies were grouped into cancer types. The numbers inside the parentheses indicate the number of publications. The group “Crk and CrkL” includes the studies in which overexpression of both Crk and CrkL was reported in a given study. The group “Crk or CrkL” includes the studies in which overexpression of Crk or CrkL expression was conducted in multiple publications. The two numbers for the group “Crk or CrkL” indicate the number of publications for Crk plus the number of publications for CrkL.

Since Nishihara et al. [66] reported overexpression of Crk in various cancer tissues, including lung, gastric, and breast cancer tissues, Crk has been reported to be overexpressed in glioblastoma [41,49,50,56], lung cancer [67], breast cancer [25], oral squamous cell carcinoma [53], bladder cancer [45], gastric cancer [68,69], and kidney cancer [56] tissues (Table 1). Elevation of CrkL protein level is also reported in non-small cell lung cancer (NSCLC) [32,70], rhabdomyosarcoma [33], breast cancer [34], gastric cancer [35,63], thyroid cancer [71], ovarian cancer [65], cervical cancer [37,72], endometrial cancer [38], pancreatic ductal adenocarcinoma [73], and colorectal cancer [47] tissues. Increased expression of both Crk and CrkL is reported in ovarian cancer [29] and breast cancer [26] tissues. In particular, both Crk and CrkL have been reported to be overexpressed in ovarian, breast, gastric, and lung cancer tissues (Figure 2).

Higher expression of Crk in cancer tissues has been reported to contribute to poor prognosis and lower survival of patients with oral squamous cell carcinoma [53], glioblastoma [50], lung cancer [64], gastric cancer [68,69], and colorectal cancer [40]. Elevated expression of CrkL in cancer tissues also has been shown to lead to poor prognosis and lower survival of patients with NSCLC [32], ovarian cancer [65], gastric [63], pancreatic ductal adenocarcinoma [73], and colorectal cancer [40]. These results together suggest that Crk and CrkL are overexpressed in many cancer types and contribute to poor prognosis.

**Figure 3:**
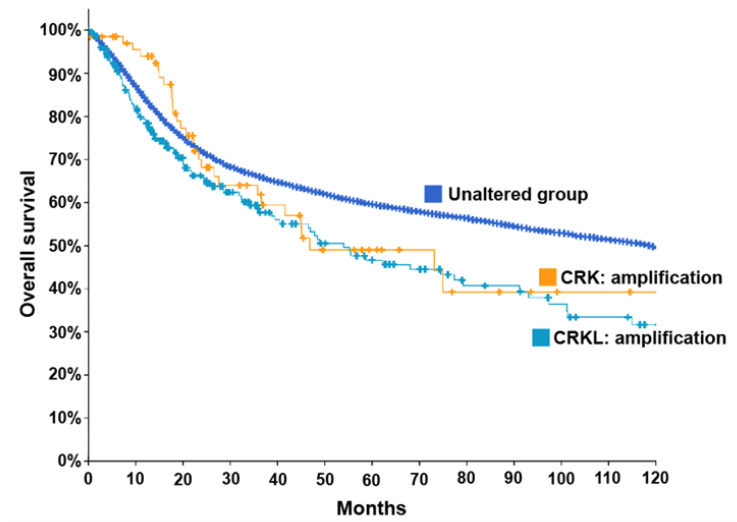
Decreased overall survival of patients with increased *CRK* and *CRKL* transcripts. The curated set of non-redundant studies in The Cancer Genome Atlas (TCGA) was analyzed by the cBioPortal [104,105] for the correlation between copy number alterations and overall survival. The total number of cases and the median months overall for each group are presented in Table 2.

Since a majority of these studies conducted immunostaining to examine protein levels in tissues, it is unclear whether the elevated levels of Crk and CrkL proteins represent elevated levels of transcripts. Analysis of the curated set of non-redundant studies in The Cancer Genome Atlas (TCGA) using the cBioPortal platform [104,105] indicates that only 0.5% and 1% of cancer patients showed copy number increases for *CRK* and *CRKL*, respectively. The results appear to be in a clear contrast to the results from the numerous studies of Crk and CrkL overexpression in cancer tissues (Table 1 and Figure 2). Although the lack of studies about a potential correlation between transcript and protein levels in various cancer types makes it difficult to draw any conclusion, it is possible that many cancers may modulate Crk and CrkL expression at the level of translation. It will be necessary to examine both copy number alterations and protein level changes in cancer tissue and study underlying mechanisms on how Crk and CrkL transcripts and proteins are upregulated in cancer. Nevertheless, lower survival was observed for the patients with amplification of *CRK* or *CRKL* transcripts (46.78 and 53.92 months for the median months overall, respectively, compared to 118.30 months with the unaltered group) (Figure 3 and Table 2). The results are consistent with the finding by Franke et al. [40]. The top 6 cancer types with 5 and higher cases of *CRK* transcript amplification include ovarian cancer, endometrial cancer, prostate cancer, melanoma, breast cancer, and sarcoma. Both ovarian and breast cancer have been reported to have Crk overexpression (Figure 2) and to be dependent on Crk for tumor cell migration and invasion (Figure 1). The top 8 cancer types with 10 and higher cases of *CRKL* transcript amplification include lung cancer, melanoma, breast cancer, ovarian cancer, soft tissue sarcoma, bladder cancer, head and neck cancer, and sarcoma. While lung, breast, and ovarian cancer have been reported to have CrkL overexpression (Figure 2), ovarian and head and neck cancer have been reported to be dependent on CrkL for tumor cell migration and invasion (Figure 1). On the other hand, heterozygous or homozygous deletion of *CRK* or *CRKL* transcripts did not show any coherent results or clear trends (Table 2). Considering the well-established, overlapping functions of Crk and CrkL in a variety of signal transduction pathways, the effects of deletion can be less evident than those of amplification. Or the numbers of patients may yet be too small to display any meaningful correlation. A similar analysis using cBioPortal of a smaller subset for lung, breast, and gastric cancer patients combined did not exhibit any apparent difference in overall survival among the patients with amplification of *CRK* and *CRKL* transcripts (data not shown). Therefore, accumulation of more datasets, together with correlation studies between copy number alterations and protein level changes in cancer tissue, would provide a more concrete explanation of whether copy number alterations of *CRK* and *CRKL* contribute to overall patient survival in individual cancer types.

## 9. Fibroblast Growth Factor Signaling and Tumor Cell Functions

Crk and CrkL are known to mediate signal transduction pathways triggered by growth factors, including fibroblast growth factor (FGF) and other extracellular stimuli [6,8]. Stimulation of endothelial cells with FGF2 induced transient phosphorylation of Crk and complex formation between FGF receptor 1 (FGFR1) and Crk, leading to endothelial cell proliferation [106]. FGF2 also stimulated phosphorylation of Crk in heparan sulfate-deficient Chinese hamster ovary 677 cells expressing FGFR1 [107]. In addition, FGF8 and CrkL genetically interacted during cardiovascular, pharyngeal, and skeletal development [21]. Furthermore, CrkL bound to FGFR1 and FGFR2 upon FGF8 treatment, and CrkL was required for FGF8-induced fibroblast survival and migration [21].

Involvement of the FGF/FGFR receptor signaling pathways in tumor cell functions has been studied in a variety of tumor cell types. Treatment of breast cancer cells with FGF2 increased cell migration by inducing aquaporin 3 expression [108]. Treatment of breast cancer cells with FGF10 increased cell migration and invasion, colony formation, and EMT marker expression [109]. FGFR2 was highly expressed in many pancreatic ductal adenocarcinoma (PDAC) tissues, and knockdown of FGFR2 IIIb and IIIc in PDAC cells inhibited cell proliferation, migration, invasion, and in vivo tumor formation [110]. FGF8 was elevated in colorectal cancer tissues, and high FGF8 expression in colorectal cancer cells led to EMT marker expression, enhanced proliferation and invasion, and in vivo tumor growth and metastasis in a YAP1-dependent manner [111]. Fibroblasts induced contact- and FGF2-dependent migration and invasion of colorectal cancer cells [112]. FGFR4 expression was elevated in nasopharyngeal carcinoma tissues, and FGFR4 knockdown reduced proliferation and migration of nasopharyngeal carcinoma cells [113]. Treatment of lung cancer cells with FGF4, but not FGF7, induced EMT, cell proliferation, migration, invasion, in vivo tumor formation, and metastasis by increasing store-operated calcium entry [114]. FGFR2 expression was elevated in gastric cancer tissues, and treatment of gastric cancer cells with FGF7 stimulated cell migration and invasion through thrombospondin upregulation [115]. FGF1 secreted from cancer-associate fibroblasts induced phosphorylation of FGFR4 and promoted ovarian cancer cell proliferation, migration, and invasion [116]. FGF8 expression is elevated in oral squamous cell carcinoma tissues, and treatment of oral squamous cell carcinoma cell lines with FGF8 promoted cell migration, invasion, and EMT [117].

These studies together demonstrate that FGF/FGFR signaling pathways promote migration and invasion of various tumor cells. Recent studies using mutant mouse models have demonstrated that Crk and CrkL play essential overlapping roles in FGF-induced lens fiber cell elongation during embryonic eye development [12] and postnatal lens development [15]. These studies clearly emphasize the importance of investigating potential contribution of Crk and CrkL to the FGF/FGF receptor (FGFR) signaling pathways. Given the roles of Crk and CrkL in various growth factor receptor-mediated signal transduction pathways, it will be important to study whether Crk and CrkL are required in the enhancement of tumor cell migration and invasion by the FGF/FGFR signaling pathways and other growth factor receptor-mediated pathways.

## 10. Summary

The contribution of Crk and CrkL to various tumor cell functions in vitro and in vivo has been studied extensively for the last two decades. A majority of the studies have focused on either Crk or CrkL and induced decreases or increases in protein levels in cells to study Crk and CrkL functions in vitro. The requirement of Crk or CrkL for cell proliferation in vitro, anchorage-independent cell growth, cell migration and invasion, in vivo tumor growth in mice, and metastasis has been demonstrated in multiple cancer cell lines. Overall, knockdown or knockout of Crk or CrkL suppressed tumor cell growth in vitro and in vivo, whereas overexpression of Crk or CrkL promoted tumor cell growth. Cancer cell migration and invasion were also inhibited by Crk or CrkL knockdown and promoted by Crk or CrkL overexpression. The effects of decreased levels of either protein on cancer cell migration and invasion contrast with the absence of effects from the loss of either protein on neuronal migration in the developing brain and T cell migration to inflamed tissues [9,11]. Interestingly, migration and invasion of glioblastoma and colorectal, pancreatic, ovarian, cervical, gastric, and liver cancer cells were dependent upon both Crk and CrkL (Table 1 and Figure 2). In addition, EMT and chemoresistance required Crk or CrkL in several cancer cells.

Immunohistochemical examination of Crk and CrkL expression in tissue specimens from cancer patients revealed that many cancers had elevated protein levels of Crk or CrkL. Ovarian, breast, gastric, and lung cancers exhibited elevated expression of both Crk and CrkL (Figure 2). Furthermore, elevated levels of Crk or CrkL proteins contributed to poor prognosis. Lung, gastric, and colorectal cancer patients showed increased protein levels for both Crk and CrkL and lower survival. The TCGA database analysis using cBioPortal also indicates that amplification of Crk or CrkL transcripts contributes to poor prognosis (Figure 3). Although these studies were instrumental in demonstrating the critical roles of Crk and CrkL in a variety of cancers, most examined either Crk or CrkL and failed to compare the roles of Crk and CrkL. Therefore, it is less clear whether Crk or CrkL alone is involved in tumor cell functions and whether both proteins play overlapping or distinct roles in each cancer type.

Fathers et al. [26] used the two breast cancer cell lines that stably expressed shRNAs for Crk and CrkL knockdown and reduced expression of CrkII, CrkI, and CrkL altogether. These cell lines exhibited significant reductions in cell migration, invasion, spreading, in vivo tumor growth, and metastasis. However, it is less clear whether knockdown of both Crk and CrkL is required for the inhibitory effects because individual knockdown of Crk or CrkL was not compared in parallel. No inhibitory effect on cell migration and less pronounced inhibitory effects on cell invasion by transient, individual knockdown of Crk and CrkL indirectly suggest potentially overlapping functions of Crk and CrkL. Recently, Franke et al. [40] induced individual and combined knockout of Crk and CrkL in colorectal cancer cells. Loss of either Crk or CrkL substantially inhibited cell migration and invasion, while loss of both completely blocked cell migration and invasion. The results indicate that both Crk and CrkL are required for colorectal cell migration and invasion. On the other hand, Park et al. [27] induced individual and combined knockdown of Crk and CrkL in a glioblastoma cell line and demonstrated a predominant role of CrkL and overlapping roles of Crk and CrkL in glioblastoma cell migration. These two recent studies elaborate on the need to study both proteins individually and combined at the same time. A clear understanding of the individual and combined functions of Crk and CrkL in each cancer type is critical to identifying the therapeutic target.

## 11. Future Directions for Therapeutic Intervention

All these studies of Crk and CrkL using cancer tissues and cancer cell lines propose Crk and CrkL as promising therapeutic targets for cancer treatment. Several reviews have previously discussed the critical roles of Crk and CrkL in cancer [8,118,119,120,121,122]. However, no apparent progress has been made in translating the information and knowledge into clinical advancement, mainly due to the absence of Crk and CrkL inhibitors. Although genetic manipulations such as gene knockout, knockdown, or overexpression helped uncover the contribution of Crk and CrkL to various tumor cell functions in vitro and in vivo, there is a significant challenge in using the genetic manipulations as therapy tools.

In addition to genetic modulation of protein levels, activities of Crk and CrkL proteins can be modulated by chemical compounds, peptides, or peptidomimetics. However, it is still unclear what represents the activity of Crk and CrkL. Since Crk and CrkL are adaptor proteins that mediate protein-protein interactions through their SH2 and SH3 domains, their interactions with target proteins may represent the activity. The challenge of this concept is that Crk and CrkL are known to interact with many proteins in various signal transduction pathways. Nevertheless, Posern et al. [123] has developed peptides that were derived from C3G and bound to SH3 domains of Crk and CrkL with high affinity and selectivity. The peptides inhibited the binding of Crk to DOCK180, to SoS, and to C3G in vitro. Kardinal et al. [124] further modified the peptide to develop cell-penetrating SH3 blocker peptides that disrupted Bcr-Abl–CrkL complexes and inhibited proliferation of primary blast cells from chronic myelogenous leukemia patients. These two studies demonstrate that peptide sequences derived from SH2- or SH3-binding proteins may be used to develop inhibitors of the binding between Crk/CrkL and their SH2 or SH3 binding partners.

More studies are needed to understand which target proteins are key binding partners in promoting tumor cell functions before assay systems are developed to test the protein-protein interactions. Inhibitors that are specific to an interaction of Crk and CrkL with a binding partner will be critical to understanding how much a given protein-protein interaction contributes to the entire function of Crk and CrkL. In order to test inhibitors, it is necessary to establish measurable, specific cellular responses mediated by Crk and CrkL. In a recent study by Park et al. [27], commercially available siRNAs were compared to identify specific and potent suppressors of Crk and CrkL using a human glioblastoma cell line. Using these siRNAs, single and double knockdowns of Crk and CrkL were induced in a glioblastoma cell line to analyze the resulting cellular phenotypes quantitatively. This study indicates that cell migration is a specific, measurable cellular outcome that requires both Crk and CrkL, providing a foundation for screening Crk and CrkL inhibitors.

Crk and CrkL have been reported to play essential overlapping functions in development [9,10,12,14] and immune cell activities [11,13]. In addition, Crk and CrkL are required in various signal transduction pathways to carry out normal cellular functions, including cytoskeletal reorganization, cell proliferation, adhesion, and migration, upon stimulation with growth factors, tyrosine kinase-coupled receptors, cytokines, and integrins. Therefore, it is necessary to carefully examine differential effects of Crk and CrkL inhibitors between tumor cells and normal cells to assess potential side effects by Crk/CrkL-targeted therapies. Since many human cancer types exhibit elevated expression of Crk and CrkL, the protein levels of Crk and CrkL in cells may be a critical deciding factor between normal and oncogenic functions of Crk and CrkL. Cancer cells are probably more prone to a reduction in protein levels or activities of Crk and CrkL. Furthermore, some oncogenic effects or signal transduction pathways may be triggered when the protein levels of Crk and CrkL exceed certain thresholds. If a certain protein-protein interaction is induced only when cells overexpress Crk and CrkL, it may be a cancer-specific event and serve as a good therapeutic target. In addition, more attention should be paid to how the synthesis and degradation of the proteins are regulated, especially in cancer cells. Considering that either Crk or CrkL has been studied in many cancer types, it is likely that cancers in different tissue types have different preferential dependences on Crk and CrkL. Therefore, developing various inhibitors, and stimulators as well, that are specific to Crk or CrkL or both will serve as essential tools in therapeutic cancer intervention and basic science research.

## Figures and Tables

**Table 1:** Classification of cancers in which Crk and CrkL were studied for various tumor cell functions.

Tumor Cell Function	Crk	CrkL	Crk and CrkL
Cell spreading	Breast [25,26] ^K^	Glioblastoma [27] ^K^	Glioblastoma [27] ^K^
In vitro cell proliferation	Ovarian cancer [28,29] ^K^ Synovial sarcoma [30] ^K^ Hepatocellular [31] ^O^	Lung [32] ^O^ Rhabdomyosarcoma [33] ^K^ Breast [34] ^K^ Gastric [35] ^K^ Cervical [36] ^K,^ [37] ^O^ Endometrial [38] ^O^ Hepatocellular [39] ^K^ Glioblastoma [27] ^K^	Glioblastoma [27] ^K^ Colorectal [40] ^K^
Anchorage-independent growth	Ovarian [28] ^K^ Glioblastoma [41] ^K^ Hepatocellular [31] ^O^	Glioma [42] ^K^ Cervical [43] ^K^	Breast [26] ^K^ Colorectal [40] ^K^
In vivo tumor growth	Ovarian [28] ^K^ Glioblastoma [41] ^K^ Breast [26,44] ^K^ Bladder [45] ^K^	Head and neck [46] ^K^ Rhabdomyosarcoma [33] ^K^ Hepatocellular [39] ^K^	
Metastasis	Breast [26] ^K^ Bladder [45] ^K^	Colorectal [47] ^K^	
Migration and invasion	Bladder [45] ^K,^ [48] ^O^ Glioblastoma [27,41] ^K,^ [49,50] ^O^ Fibrosarcoma [51] ^O^ Breast/cervical/lung [25] ^K^ Ovarian [28,29] ^K^ Synovial sarcoma [52] ^K^ Oral squamous [53] ^K^ Prostate [54,55] ^K^ Breast [26] ^K,^ [56] ^O^ Lung [57,58] ^O^ Gastric [59] ^K^ Colorectal [40] ^K^ Hepatocellular [31] ^O^ Pancreatic [60] ^K^	Head and neck [46] ^K^ Glioma [42] ^K^ Ovarian [61] ^K^ Hepatocellular [62] ^O^ Gastric [63] ^K^ Cervical [43] ^K,^ [37] ^O^ Colorectal [40] ^K^ Glioblastoma [27] ^K^	Colorectal/pancreatic [40] ^K^ Glioblastoma [27] ^K^
Epithelial-mesenchymal transition	Bladder [45] ^K^ Lung [64] ^O^ Breast [44] ^K^ Colorectal [40] ^K^	Ovarian [65] ^K^ Gastric [63] ^K^ Colorectal [40] ^K^	Colorectal/pancreatic [40] ^K^
Chemoresistance		Cervical [37] ^O^ Endometrial [38] ^O^	Colorectal [40] ^K^
Overexpression in cancer tissues	Lung/gastric/breast [66] Glioblastoma [41,50,56] Lung [67] Breast [25] Oral squamous [53] Bladder [45] Gastric [68,69] Kidney [56]	Lung [32,70] Rhabdomyosarcoma [33] Breast [34] Gastric [35,63] Thyroid [71] Ovarian [65] Cervical [37,72] Endometrial [38] Pancreatic [73] Colorectal [47]	Ovarian [29] Breast [26]
Lower survival	Oral squamous [53] Glioblastoma [50] Lung [64] Gastric [68,69] Colorectal [40]	Lung [32] Ovarian [65] Gastric [63] Pancreatic [73] Colorectal [40]	Colorectal [40]

Studies in which Crk or CrkL was studied for the indicated tumor cell function are classified into cancer types and listed by year of publication of the earliest paper. References with the initial K indicate studies in which Crk or CrkL expression was decreased by gene knockdown or knockout. References in the initial O indicate studies in which Crk or CrkL was overexpressed. Despite an effort to be inclusive in choosing the references for citation, a few publications were excluded due to unclear results.

**Table 2:** Overall survival of patients with gain or loss of Crk or CrkL transcripts.

Copy Number Alteration	Total Number of Cases	Median Months Overall
Unaltered group	21,270	118.30
CRK: amplification	69	46.78
CRKL: amplification	231	53.92
CRK: heterozygous deletion	5072	81.93
CRK: homozygous deletion	108	94.00
CRKL: heterozygous deletion	3542	71.17
CRKL: homozygous deletion	51	158.00

The curated set of non-redundant studies in The Cancer Genome Atlas (TCGA) was analyzed for copy number alterations by the cBioPortal [104,105]. The following Onco Query Language (OQL) queries were used in combinations to identify copy number alterations of Crk and CrkL: CRK: AMP, CrkL: AMP, CRK: HETLOSS, CRK: HOMDEL, CRKL: HETLOSS, and CRKL: HOMDEL. Depending on the OQL combination, the unaltered group ranged from 21,248 to 21,270 for the total number of cases and from 118.30 to 118.60 for the median months overall because of variations due to exclusion of overlapping samples and patients.

## Data Availability

The data supporting the findings of this study are contained in the article. The data that are described but not shown in the article will be shared upon request by the author.

## Abbreviations

Crk	CT10 regulator of kinase
CrkL	Crk-like
GBM	Glioblastoma
EMT	Epithelial-mesenchymal transition
TCGA	The Cancer Genome Atlas
OQL	Onco Query Language
NSCLC	Non-small cell lung cancer
FGF	Fibroblast growth factor
FGFR	Fibroblast growth factor receptor
